# Evaluation of drug-drug interaction between Suraxavir Marboxil (GP681) and itraconazole, and assessment of the impact of gene polymorphism

**DOI:** 10.3389/fphar.2024.1505557

**Published:** 2025-04-11

**Authors:** Mai Han, Gang Cui, Yan Zhao, Xianbo Zuo, Xiaoxue Wang, Xin Zhang, Na Mi, Jiangli Jin, Chunyan Xiao, Jing Wang, Wei Wu, Yajuan Li, Jintong Li

**Affiliations:** ^1^ Drug Clinical Trial Research Center, China-Japan Friendship Hospital, Beijing, China; ^2^ Qingfeng Pharmaceutical Group Co., Ltd., Ganzhou, Jiangxi, China; ^3^ Department of Pharmacy, State Key Laboratory of Respiratory Health and Multimorbidity, China-Japan Friendship Hospital, Beijing, China

**Keywords:** DDI (drug-drug interaction), itraconazole, Suraxavir Marboxil (GP681), safety, genetic polymorfism, pharmacokinetic

## Abstract

**Introduction:**

Suraxavir Marboxil (GP681) is a prodrug metabolized to GP1707D07, which inhibits influenza viral replication by targeting cap-dependent endonuclease through a single oral dose. This study assesses the in vivo drug-drug interaction (DDI) potential between GP681 (including its major metabolite GP1707D07, a substrate of CYP3A4) and itraconazole in healthy Chinese subjects, along with the safety profiles during co-administration. Additionally, it evaluates the impact of CYP1A2, CYP2C19, and CYP3A4 gene polymorphisms on GP1707D07 metabolism.

**Methods:**

The study enrolled twelve healthy adult subjects to receive the treatments consisting of GP681 monotherapy and GP681-itraconazole co-administration in a fixed-sequence. Single nucleotide polymorphisms (SNPs) in CYP gene loci were also analyzed.

**Results:**

Co-administration of itraconazole increased the GP1707D07 AUC0- ∞ by about 2.5 folds and Cmax by about 1.4 folds compared with GP681 administered alone. Differences in system exposure were more pronounced during the terminal elimination phase than the early stage of GP1707D07 metabolism. No significant increase in adverse events was observed during co-administration. Using random forest algorithm, we estimated effects of cytochrome P450 enzymes followed the order of CYP 3A4 > CYP 1A2 > CYP 2C19. We also hypothesized CYP 3A4 rs4646437 A>G, CYP 3A4 rs2246709 G>A, and CYP 2C19 rs12768009 A>G to be mutations that enhanced enzyme activity, while CYP1A2 rs762551 C>A weakened it.

**Discussion:**

The pharmacokinetic changes of GP1707D07 during itraconazole co-administration are insufficient to warrant clinical action. Random forest algorithm enhances the understanding of pharmacogenetic variants involved in GP1707D07 metabolism and may serve as a potent tool for assessing gene polymorphism data in small clinical samples.

**Clinical Trial Registration:**

clinicaltrials.gov, identifier NCT05789342.

## 1 Introduction

Influenza A virus (IAV) and influenza B virus (IBV) are among the most prominent human respiratory pathogens. About 3–5 million severe cases of influenza are associated with 300,000–650 000 deaths per year globally. Effective antivirals that reduce morbidity and mortality constitute a crucial component of the first line of defense against influenza. There is unmet medical need not only for the treatment of seasonal influenza but also as a treatment option for future pandemics (Caceres et al., 2022; Jones et al., 2023; Zhao et al., 2024). Mutations associated with antiviral resistance are common and highlight the need for further improvement and development of novel anti influenza drugs (Caceres et al., 2022). IAV and IBV are enveloped viruses containing 8 segments of single strand negative-sense genomic RNA in the form of viral ribonucleoprotein particles (vRNPs) associated with the polymerase complex (PB1, PB2, PA) and the nucleoprotein (NP) (Caceres et al., 2022; Krammer et al., 2018). Transcription of viral RNAs depends on a “cap snatching” mechanism that utilizes the cap-binding activity of PB2 and the cap-dependent endonuclease (CEN) in the PA subunit (Krammer et al., 2018). Structural studies revealed that specific residues in the N-terminus of PA were highly conserved and mutations in these residues resulted in the loss of endonuclease activity (Zhao et al., 2024; O'Hanlon and Shaw, 2019; Dias et al., 2009; Kuo et al., 2021; Liu et al., 2021; De Clercq, 2006). As a result, cap-dependent endonuclease (CEN) inhibitor targeting the PA polymerase subunit are of special clinical interest (Caceres et al., 2022; Zhao et al., 2024; O'Hanlon and Shaw, 2019; Kuo et al., 2021; Liu et al., 2021; De Clercq, 2006; Noshi et al., 2018; Takashita et al., 2018; Omoto et al., 2018; Ikematsu et al., 2020).

**FIGURE 1:**
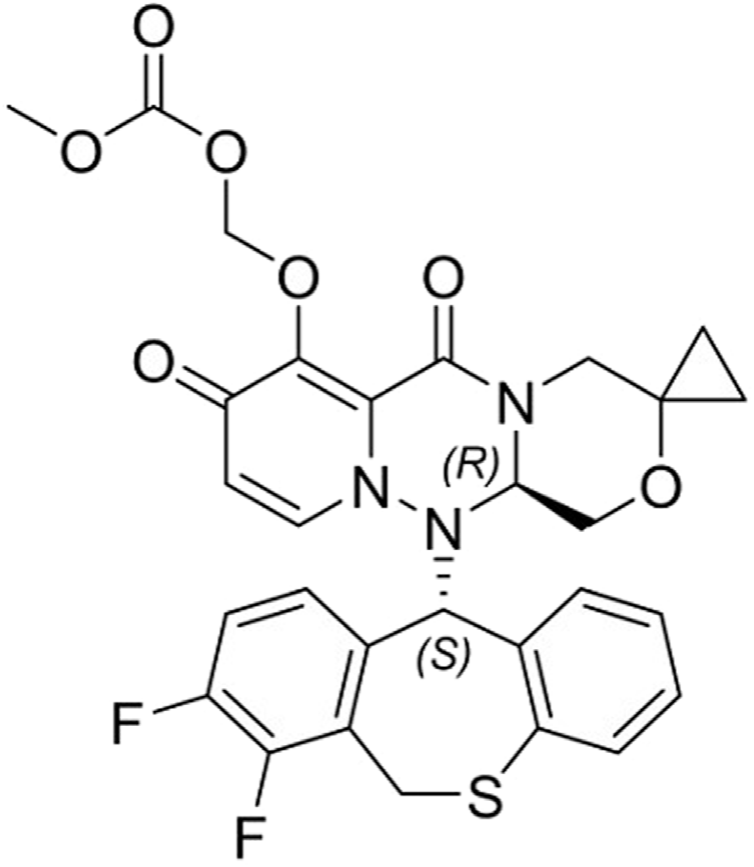
Chemical structure of Suraxavir Marboxil (GP681).

Suraxavir Marboxil (GP681) [(((R)- 12'- ((S)- 7, 8- difluoro- 6, 11- dihydro- 2- benzothiophene- 11- yl)- 6′, 8′- dioxide- 6′, 8′, 12′, 12a′- tetrahydro- 1′H-, 4′H- spiro [cyclopropane- 1, 3'- [1, 4] oxazine [3, 4-c] pyridine and [2,1-f] [1, 2, 4] triazine]- 7′- yl) oxy) methyl carbonate, Figure 1) developed by Qingfeng Pharmaceutical Group Co., Ltd., is an antiviral prodrug that is metabolized to a small molecule active form (GP1707D07). The metabolite GP1707D07 is a polymerase acidic protein inhibitor, which selectively inhibits the cap-dependent endonuclease of influenza virus, preventing the replication of the virus. *In vitro* experiments have shown that GP1707D07 has inhibitory activity against all tested avian influenza virus strains, with better *in vitro* antiviral activity (in terms of average EC_50_ and EC_90_) than baloxavir (unpublished data).

Preclinical studies have shown that following oral administration, Suraxavir Marboxil (GP681), the prodrug is scarcely detected in plasma but converted to an active form GP1707D07 by carboxylesterase CES2. GP1707D07 exhibits a high plasma protein binding rate, and is widely distributed across various tissues without accumulating in any tissues at extremely high concentrations or for prolonged durations. GP1707D07 is mainly metabolized by cytochrome P450 (CYP) 1A2, CYP 2C8, and CYP 3A4, with varying degrees of involvement from CYP2B6, CYP2C9, CYP2D6, and CYP2C19. Neither Suraxavir Marboxil (GP681) nor its metabolite, GP1707D07, serves as substrates for the BCRP and MDR1 transporters. They do not significantly inhibit or induce liver enzymes, nor do they inhibit the transport activity of transporters BCRP, MDR1, OATP1B1, OATP1B3, OAT1, OAT3, and OCT2. Suraxavir Marboxil (GP681) and GP1707D07 are mainly excreted in feces, with a small amount excreted in urine and bile. Mass balance study confirmed GP1707D07 to be the major radioactive substance, accounting for approximately 95.4% of the total radioactivity in serum, and total radioactivity recovery rate was 90.1% (84.1% in feces and 6.0% in urine) within 336 h after the oral administration of Suraxavir Marboxil (GP681) (unpublished data).

Phase I study has shown the median time to peak concentration (T_max_) of GP1707D07 to be 3.0 h–4.5 h and the half-life (T_1/2_) to be 58 h–76 h. Systemic GP1707D07 exposure demonstrated generally dose-proportional increases in the fasted state. In addition, for all tested dose cohorts (20 mg–80 mg), the C_24_ of GP1707D07 exceeded the estimated clinically effective concentration (6.87 ng/mL), and could maintain above this target concentration by 72–136 h.

Itraconazole is a broad spectrum triazole antifungal agent primarily metabolized by CYP3A4. Itraconazole and its main metabolite hydroxyitraconazole are potent inhibitors of CYP3A4, defined as a midazolam area under the curve ratio [mAUCR] approximately ≥5 cutoff (Piérard et al., 2000; Liu et al., 2016; Ke et al., 2014; Heykants et al., 1989; Templeton et al., 2008). As a result, itraconazole can inhibit the metabolism of drugs metabolized by CYP3A4 and has been widely used as an inhibitor in drug-drug interaction (DDI) studies. When itraconazole is administrated in combination with drugs of CYP3A4-meditated metabolism, the concentrations of the drug itself and/or its active metabolites may increase, potentially leading to significant clinical implications such as increased or prolonged therapeutic effects and adverse reactions.

Preclinical *in vitro* studies have shown that GP1707D07, the active metabolite of Suraxavir Marboxil (GP681), is a substrate of CYP3A4. According to the “Technical Guidelines for Drug Interaction Studies” issued by the Central for Drug Evaluation (CDE) in 2021 (Technical Guidelines for Drug Interaction Studies, 2024) and the ICH “M12: Drug Interaction Studies” guideline (Ich M12 on Drug Interaction Studies, 2024), this study selected itraconazole as a CYP3A4 inhibitor. The primary objective of this study was to assess *in vivo* DDI potentials between Suraxavir Marboxil (GP681) (as well as its major metabolite GP1707D07) and itraconazole in healthy Chinese subjects. The secondary objective was to assess the safety profiles of oral Suraxavir Marboxil (GP681) tablets in combination with itraconazole capsules.

Furthermore, as an exploratory research, the study was also aimed to evaluate the impact of CYP1A2, CYP2C19, and CYP3A4 gene polymorphisms on the metabolism of Suraxavir Marboxil (GP681). Random forest is an ensemble algorithm in machine learning known as Bagging (Bootstrap AGgregation), which is a classifier that consists of multiple decision trees. The advantages of random forest include not requiring feature selection, the ability to assess feature importance, resistance to overfitting, and maintaining accuracy even with a significant portion of features missing (Ho, 1995; Breiman, 2001; Hu and Szymczak, 2023; Jin et al., 2023; Shi et al., 2023; Tian et al., 2023; Becker et al., 2023; Huynh-Thu and Geurts, 2019). Using the random forest algorithm, we assessed the impact of gene polymorphisms in CYP1A2, CYP2C19, and CYP3A4 on the metabolism of GP1707D07, both in GP681 monotherapy and in GP681-itraconazole co-administration. The results provided valuable insights into the pharmacogenetic variants involved in the metabolism of GP1707D07.

## 2 Materials and methods

### 2.1 Study design

**FIGURE 2:**
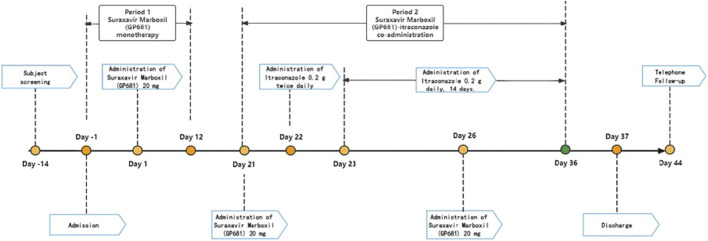
Design of the drug-drug interaction study between Suraxavir Marboxil (GP681) and itraconazole.

This single-center, open-label, self-controlled, fixed-sequence study, sponsored by Qingfeng Pharmaceutical Group Co., Ltd., was conducted at a single clinical research site in the China–Japan Friendship Hospital (Beijing, China). Each subject received the following treatment: Suraxavir Marboxil (GP681) 20 mg in the fasted state on day 1 and day 26. Itraconazole capsules (Sporanox^®^: Xi’an Yangsen Pharmaceutical Co., Ltd., China) 0.2 g twice daily (BID, *bis in die*) on day 22. Itraconazole 0.2 g daily from day 23 to day 36 [including day 26, when itraconazole was co-administrated with Suraxavir Marboxil (GP681)]. The washout period is 21 days (Figure 2). It should be noted that in the following sections, when discussing the PK profile, this study was divided into Period 1 (Suraxavir Marboxil (GP681) monotherapy, from day −1 to day 12), and Period 2 (Suraxavir Marboxil (GP681)-itraconazole co-administration, from day 21 to day 36). When discussion the safety profile, this study was divided into Stage 1 (Suraxavir Marboxil (GP681) monotherapy stage, from day 1 to day 21), Stage 2 (itraconazole monotherapy stage, from day 22 to day 25), and Stage 3 (Suraxavir Marboxil (GP681)-itraconazole co-administration stage, from d 26 to d 36).

As observed in previous Phase I and Phase II clinical trials of Suraxavir Marboxil (GP681), the regimen of oral dose 20 mg is estimated to be the minimal clinically effective dose. The recommended dose of Suraxavir Marboxil (GP681) for Phase III clinical trial is 40 mg. Considering that itraconazole is a strong CYP3A4 inhibitor, we selected the dosage of Suraxavir Marboxil (GP681) 20 mg in this DDI study, in case of unexpected increased exposure of Suraxavir Marboxil (GP681) and/or its active metabolites after co-administration.

### 2.2 Ethics

The study protocol and informed consent form were approved by the Clinical Research Ethics Committee of the China–Japan Friendship Hospital (Clinical Trial registration identifier: NCT05789342). The study was conducted in accordance with the principles of Good Clinical Practice and the Declaration of Helsinki. Written informed consent had been obtained from all the subjects before enrollment.

### 2.3 Subjects

Subjects were male or female healthy volunteers. Key inclusion criteria required subjects to be aged 18–55 years, with body mass index (BMI) 19.0–28.0 kg/m^2^, and a minimum body weight 50 kg for males or 45 kg for females. Key exclusion criteria included: 1) History of allergic conditions or allergic diseases, or a history of allergic reactions attributed to drugs. 2) Subjects with severe infection, trauma, gastrointestinal surgery, or other major surgical operations within 6 months before screening. 3) Received any drugs that inhibit or induce the CYP450 enzyme (i.e., phenytoin, rifampin, carbamazepine, fluvoxamine, enoxacin, ticlopidine, gemfibrozil, clopidogrel, clarithromycin, itraconazole, ketoconazole, ritonavir) 4 weeks prior to the screening period. 4) Received any drugs (including Chinese herbal medicine, vitamins, and supplements) within 14 days prior to dosing.

### 2.4 Blood sample collection

Blood samples were collected at the following time points: pre–dose, 0.5, 1, 2, 3, 4, 5, 6, 8, 12, 24, 36, 48, 72, 120, 168, and 264 h post-dose on day 1 and day 26. In addition, a blood sample were collected before dosing on day 1 for genetic polymorphism assessment.

### 2.5 Sample analysis and pharmacokinetic analysis

A validated high-performance liquid chromatography-tandem mass spectrometry (HPLC-MS/MS) method was used to determine plasma concentrations of Suraxavir Marboxil (GP681) and its active metabolite GP1707D07 at different times, with the quantification range of 0.3/0.3–300/300 ng/mL for Suraxavir Marboxil (GP681) and GP1707D07. The lower limit of quantification (LLOQ) for both compounds was 0.300 ng/mL. Phoenix^®^ WinNonlin^®^ 8.3 was used to estimate and analyze the pharmacokinetic parameters of non-compartmental model from plasma concentration data. Pharmacokinetic parameters included: maximum plasma concentration (C_max_), area under the plasma concentration-time curve extrapolated from time zero to infinity (AUC_0- ∞ _), time to C_max_ (T_max_), half-life (t_1/2_), λz, AUC__%Extrap_, V_z_/F, CL/F, and mean residence time (MRT).

### 2.6 Genotyping analysis

Peripheral blood samples were used as a source of DNA for CYP genotyping. Single nucleotide polymorphisms (SNPs) in 8 CYP1A2, 11 CYP2C19 and 13 CYP3A4 gene loci were analyzed in this study. Genotyping for SNP was detected in BioMiao Biological Technology (Beijing) Co., Ltd. MassARRAY iPLEXassay system was used for target SNP genotyping. which is a flight mass spectrometry-based genotyping technology developed by Agena Bioscience, Inc. (Agena, San Diego, United States). This technology is a flight mass spectrometry-based genotyping technology developed by Agena Bioscience, Inc. (Agena, San Diego, United States), it amplifies the DNA sequence containing SNP sites through PCR amplification and then amplifies the above PCR products through specific single-base extension primers. In the ddNTP reaction system, the extension primers only amplify the complementary bases with the SNP sites to be tested, which terminates. The final extended product was analyzed using a time-of-flight mass spectrometry (TOF) system, and SNPs were discriminated based on the molecular weight differences of different alleles. Haplotypes were defined by the SNPs in each gene.

### 2.7 Safety assessment

Safety was assessed through physical examination, vital sign measurements, clinical laboratory tests, 12-lead electrocardiograms, and assessment of self-reported symptoms. Adverse events were collected from the first dose through the end of the study. All safety data collected were assessed for severity and relationship to each study drug, Suraxavir Marboxil (GP681) or itraconazole. The severity of an adverse event was graded according to Common Terminology Criteria for Adverse Events (CTCAE) v5.0. For each adverse event occurred, investigators continued to follow up until it was resolved. Adverse event analysis is based on Safety Set (SS), coded with MedDRA version 26.0. Adverse events will be summarized by system organ class (SOC) and preferred term (PT) in terms of number of cases, occurrences, and incidence rate.

### 2.8 Statistics

Statistical analysis was performed using SAS (version 9.4 or above) software programming. Pharmacokinetic concentration analysis was based on Pharmacokinetic Analysis Concentration Set (PKCS). Individual and mean concentrations (mean ± SD) were plotted against planned blood sampling time points for the analyte on linear and semi-log scales. Pharmacokinetic parameter analysis was based on Pharmacokinetic Analysis Parameter Set (PKPS). Drug interaction analysis was based on Drug Interaction Set (DIS). The geometric mean ratios and their 90% confidence intervals of pharmacokinetic parameters C_max_, AUC_0-t_, and AUC_0- ∞_ under Suraxavir Marboxil (GP681) single dose and Suraxavir Marboxil (GP681)-itraconazole co-administration conditions were calculated after natural logarithm transformation, using a linear mixed effects model with subjects as random effects and treatment (combined/single) as fixed effects. If the 90% confidence intervals of the geometric mean ratios of C_max_, AUC_0-t_, and AUC_0- ∞_ under combined/single drug administration conditions fall within the range of 80.00%–125.00%, it can be considered that there is no significant difference in pharmacokinetics.

### 2.9 Random forest modeling

Using the random forest module of SPSS (Version 24.0) online, we excluded 18 SNPs that were wild-type (0/0) in all 12 subjects (see *Results* section), and set the following groups of independent variables (x) and dependent variables (y) for analysis. (1) x = SNPs of CYP 1A2, CYP 2C19, CYP 3A4. y = CL/F of GP1707D07 in Period 1. (2) x = SNPs of CYP 1A2, CYP 2C19. y = the retention ratio of GP1707D07 CL/F in Period 2 compared to Period 1, calculated as follows:

$$\text{GP}1707D07\text{ CL}/F\text{ retention ratio}=\frac{\;\text{GP}1707D07\;\text{CL}/F\;Period\;2}{\text{GP}1707D07\;\text{CL}/F\;Period\;1}$$

(3) x = SNPs of CYP 3A4. y = the decrease ratio of GP1707D07 CL/F in Period 2 compared to Period 1, calculated as follows:

$$\text{GP}1707D07\text{ CL}/F\text{ reduction ratio}=\frac{\text{GP}1707D07\;\text{CL}/F\;Period\;1-\text{CL}/F\;Period\;2}{\text{GP}1707D07\;\text{CL}/F\;Period\;1}$$

The task type is a regression task, with a training set ratio of 0.8.

## 3 Results

### 3.1 Subject disposition and demographics

A total of 12 subjects were enrolled in this DDI study. There were 6 male and 6 female subjects, all of Han ethnicity, with an average age of 34.3 years and an average weight of 62.3 (±10.8) kg, as shown in Table 1. All subjects completed this study. The actual doses of Suraxavir Marboxil (GP681) and itraconazole they received was consistent with the planned dose.

**TABLE 1:** Subject demographics in this drug–drug interaction study between GP681 and itraconazole.

Demographic N = 12	
Age (years), mean (SD)	34.3 (6.47)
Weight (kg), mean (SD)	62.313 (10.7699)
Height (cm)	165.06 (12.582)
BMI (kg/m^2^), mean (SD)	22.73 (1.741)
Sex, male, n (%)	6 (50.0)
Ethnicity not Han Chinese, n (%)	0

### 3.2 Pharmacokinetic parameter analysis

For Suraxavir Marboxil (GP681), only 4 blood samples from 4 subjects had Suraxavir Marboxil (GP681) concentrations above the quantification limit (0.3 ng/mL), collected at Day 26, 0.5 h post-dose of Suraxavir Marboxil (GP681)-itraconazole co-administration. The highest plasma concentration of Suraxavir Marboxil (GP681) was 0.473 ng/mL (ID 305). In all the rest of blood samples, plasma concentrations of Suraxavir Marboxil (GP681) were below the quantification limit. Therefore, the calculation of pharmacokinetic parameters of Suraxavir Marboxil (GP681) and the statistical analysis were not performed. For GP1707D07, all samples had concentrations above the quantification limit, with good reproducibility in biological sample analysis.

**FIGURE 3:**
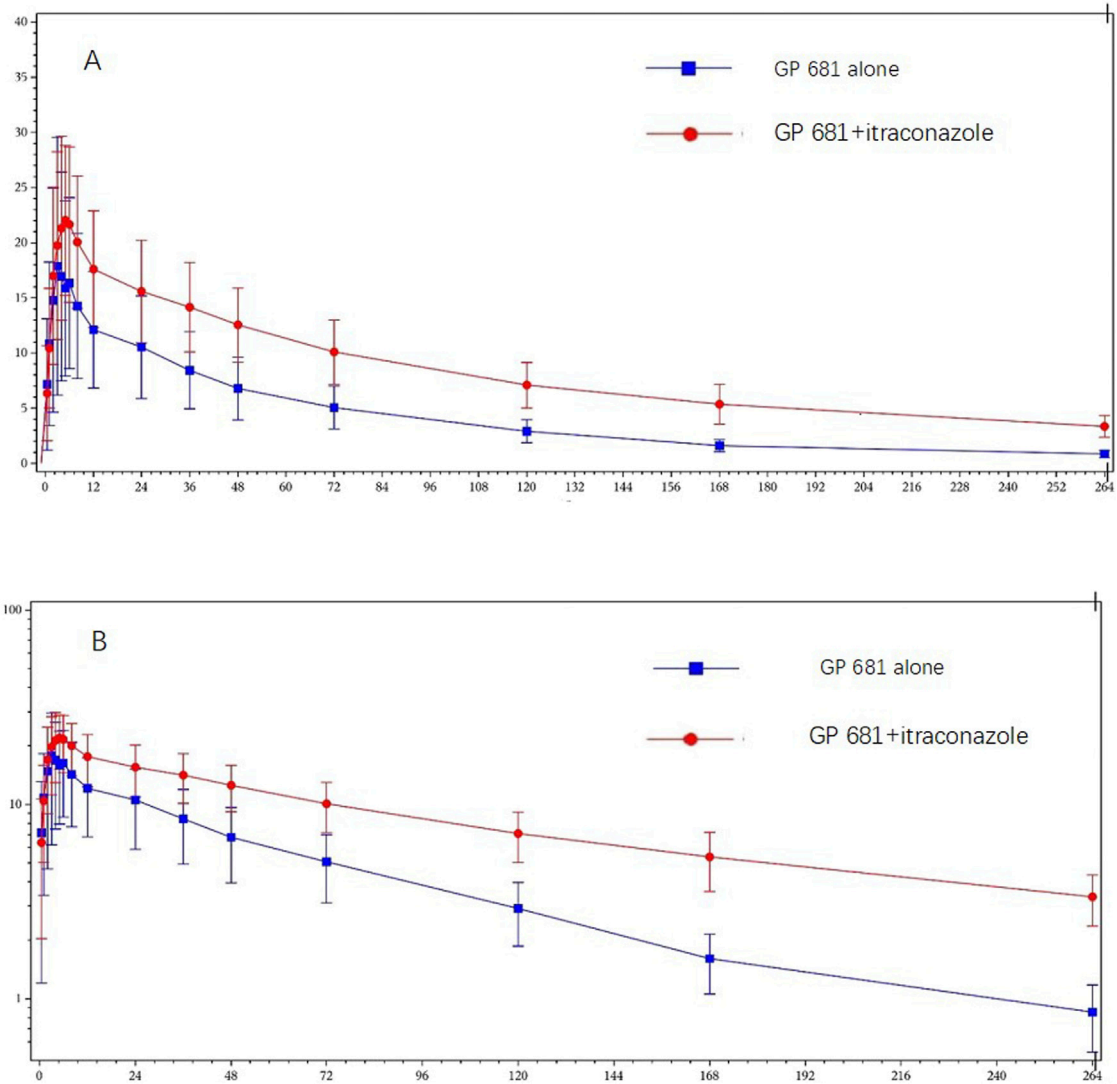
Plasma concentration profiles of GP1707D07 in GP681 monotherapy or GP681-itraconazole co-administration. Mean (+standard deviation) plasma concentration profiles were plotted with linear scale **(A)** and semi-log scale **(B)**.

Compared with Suraxavir Marboxil (GP681) administrated alone, when Suraxavir Marboxil (GP681) was co-administered with itraconazole, the peak plasma concentration time (t_max_) of GP1707D07 was slightly prolonged (4.5 vs. 4.0 h), the half-life (t_1/2_) was significantly prolonged (107.9 vs. 65.8 h), and the mean residence time (MRT_0- ∞ _) was approximately doubled (172.4 vs. 88.7 h). C_max_, AUC_0-t_ and AUC_0- ∞_ of GP1707D07 increased by 22%, 107%, and 153% in Period 2 compared to those in Period 1, respectively (Figure 3; Table 2).

**TABLE 2:** Summary of pharmacokinetic parameters of GP1707D07 following administration of GP681 monotherapy and GP681-itraconazole co-administration.

PK parameter	Period 1 GP681 monotherapy (N = 12)	Period 2 GP681-itraconazole co-administration (N = 12)^a^	Period 2 GP681-itraconazole co-administration (N = 12)^b^
C_max_ (ng/mL)	19.3 (11.2)	23.5 (8.1)	—^b^
AUC_0-t_ (h*ng/mL)	1,040.1 (410.2)	2,151.5 (621.4)	—^b^
AUC_0- ∞ _ ^a^(h*ng/mL)	1,122.9 (429.5)	2,844.7 (624.5) (N = 4)	2,744.9 (802.8)
AUC__%Extrap_ ^a^ (%)	7.9 (3.4)	17.1 (7.8)	22.3 (4.8)
t_max_ ^c^ (h)	4.0 (2.0–6.0)	4.5 (3.0–6.0)	—^b^
t_1/2_ ^a^(h)	65.8 (12.1)	107.9 (11.4) (N = 4)	127.2 (21.8)
λ_z_ ^a^ (h^−1^)	0.011 (0.0021)	0.007 (0.0008) (N = 4)	0.006 (0.0009)
CL/F^a^ (L/h)	21.1 (10.6)	7.4 (2.0) (N = 4)	8.0 (2.9)
V_z_/F^a^ (L)	2071.2 (1,333.0)	1,122.4 (175.2) (N = 4)	1,442.2 (537.3)
MRT_0-t_ (h)	67.2 (7.5)	91.9 (4.4)	—^b^
MRT_0- ∞ _ (h)	88.7 (17.0)	172.4 (26.7)	—^b^

Data are mean ± SD, except for t_max_ which are presented as median and range.

N, number of subjects; AUC_0-t_, area under the concentration-time curve (AUC) from time 0 to the last quantifiable concentration; AUC_0- ∞ _, AUC, extrapolated to infinity; C_max_, maximum observed plasma concentration; CL/F, apparent total plasma clearance; t_max_, time to maximum concentration); t_1/2_, apparent terminal elimination half-life; V_z_/F, apparent volume of distribution during the terminal elimination phase.

^a^ Data in 8 subjects were not included in the descriptive statistical analysis, because their %AUC_extrap_ (percentage of AUC, that is due to extrapolation from the last measurable concentration to infinity) were >20%.

^b^ This column includes data from all 12 subjects. Data identical to left are not repeated here.

^c^ Median (range).

The geometric mean ratios and their 90% confidence intervals of C_max_, AUC_0-t_, and AUC_0- ∞_ were 137.7% (98.5%, 192.3%), 215.2% (170.7%, 271.3%), and 255.4% (204.5%, 319.0%), respectively. None of them fall completely within the most conservative range of 80.00%–125.00%, indicating that co-administration of itraconazole with Suraxavir Marboxil (GP681) tablets significantly increases the exposure of GP1707D07 (Table 3).

**TABLE 3:** Comparison of pharmacokinetic parameters of GP1707D07 using point estimate on geometric mean following administration of GP681 alone or GP681-itraconazole co-administration.

Parameter	Geometric mean		Point estimate of geometric mean (90% CI) Period 2/Period 1
	Period 1 GP681 monotherapy (N = 12)	Period 2 GP681-itraconazole co-administration (N = 12)	
GP1707D07			
C_max_ (ng/mL)	16.0	22.0	137.7 (98.5, 192.3)
AUC_0-t_ (h*ng/mL)	995.8	2057.0	215.2 (170.7, 271.3)
AUC_0- ∞ _ (h*ng/mL)	1,038.7	2,652.9	255.4 (204.5, 319.0)

CI, confidence interval.

### 3.3 Genetic polymorphism analysis

The following 18 SNPs were found to be wild-type (0/0) in all 12 subjects, including 5 CYP1A2 loci (rs2069526 T/T, rs4646425 C/C, rs72547516 A/A, rs4646427 T/T, rs72547517 G/G), 5 CYP2C19 loci (rs11188072 C/C, rs12248560 C/C, rs28399504 A/A, rs41291556 T/T, rs56337013 C/C), and 8 CYP3A4 loci (rs4986910 A/A, rs28371759 A/A, rs355599367 G/G, rs55951658 T/T, rs56324128 C/C, rs2740574 T/T, rs62471956 G/G, rs472660 G/G). The genotype distribution of the other loci showed differences (Table 4).

**TABLE 4:** Allele frequencies of 14 SNPs in this drug–drug interaction study between GP681 and itraconazole.

Cytochrome	SNP ID	Genotype	Frequency (%)
CYP 1A2	rs762551	A/A	41.67
		A/C	50.00
		C/C	8.33
	rs2472304	G/G	66.67
		G/A	33.33
	rs2470890	C/C	66.67
		C/T	33.33
CYP 2C19	rs3814637	C/C	83.33
		C/T	16.67
	rs11568732	T/T	83.33
		T/G	16.67
	rs12768009	G/G	25.00
		G/A	66.7
		A/A	8.30
	rs12769205	A/A	33.33
		A/G	66.67
	rs4986893	G/G	83.33
		G/A	16.67
	rs4244285	G/G	33.33
		G/A	66.67
CYP 3A4	rs3735451	T/T	41.67
		T/C	50.00
		C/C	8.33
	rs4646440	G/G	50.00
		G/A	33.33
		A/A	16.67
	rs2242480	C/C	50.00
		C/T	33.33
		T/T	16.67
	rs4646437	G/G	66.67
		G/A	33.33
	rs2246709	A/A	50.00
		A/G	50.00

Note. 18 SNPs, that were found to be wild-type (0/0) in all 12 subjects are not listed in this table. (See in the main text).

**FIGURE 4:**
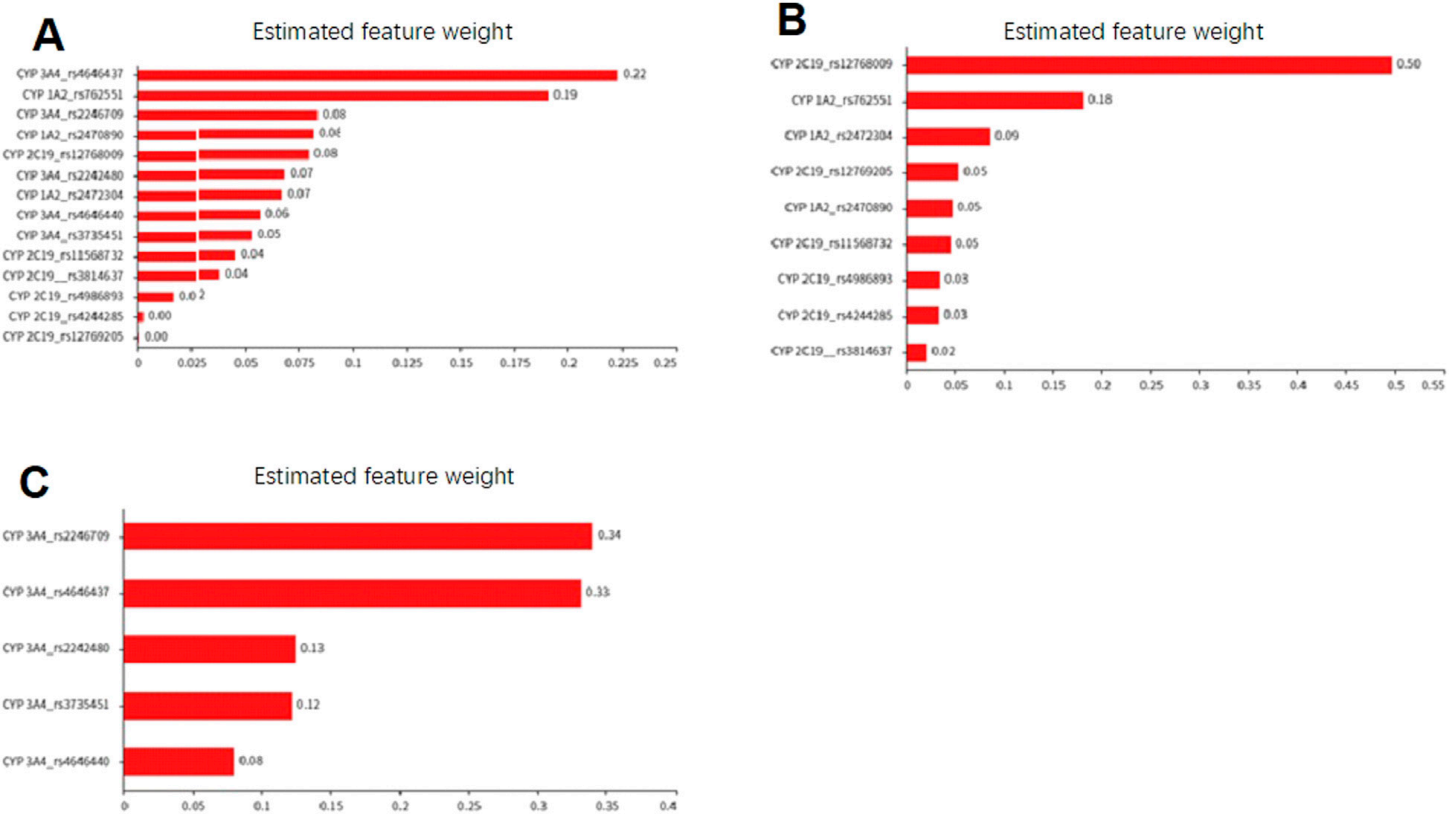
Feature weight diagram based on the random forest model to evaluate the impact of cytochrome P450 polymorphism on the metabolic capacity of GP1707D07. **(A)** Effect of SNPs of CYP 1A2, CYP 2C19, and CYP 3A4 on CL/F of GP1707D07 in Period 1. **(B)** Effect of SNPs of CYP 1A2 and CYP 2C19 on the retention ratio of GP1707D07 CL/F in Period 2 compared to Period 1. **(C)** Effect of SNPs of CYP 3A4 on the reduction ratio of GP1707D07 CL/F in Period 2 compared to Period 1.

Using the random forest algorithm for evaluation, the total weight value of the CYP 3A4 gene locus in the Suraxavir Marboxil (GP681) monotherapy phase was approximately 48%, while CYP 1A2 and CYP 2C19 was 34% and 18%, respectively. The gene locus rs4646437 of CYP 3A4 had the highest feature weight value, accounting for 22%, playing a key role in model construction. It was followed by the CYP 1A2 gene locus rs762551, with a feature weight value of 19%. Among the gene loci of CYP 2C19, only rs12768009 had a relatively high weight (8%), while the gene polymorphisms of other CYP 2C19 loci had minor impact on the metabolism of GP1707D07 (feature weight values of 0.1%–4%) (Figure 4; Table 5).

**TABLE 5:** Estimated feature weight of cytochrome P450 SNPs in GP1707D07 metabolism based on the random forest model.

Independent variable (x)	SNP ID	CYP 1A2, CYP 2C19, and CYP 3A4 SNPs	CYP 1A2, and CYP 2C19 SNPs	CYP 3A4 SNPs
Dependent variable (y)		CL/F in Period 1	CL/F retention ratio	CL/F reduction ratio
Feature weight value				
CYP 1A2	rs762551	0.19	0.18	NA
	rs2472304	0.07	0.09	NA
	rs2470890	0.08	0.05	NA
CYP 2C19	rs3814637	0.04	0.02	NA
	rs11568732	0.04	0.05	NA
	rs12768009	0.08	0.50	NA
	rs12769205	0.00	0.05	NA
	rs4986893	0.02	0.03	NA
	rs4244285	0.00	0.03	NA
CYP 3A4	rs3735451	0.05	NA	0.12
	rs4646440	0.06	NA	0.08
	rs2242480	0.07	NA	0.13
	rs4646437	0.22	NA	0.33
	rs2246709	0.08	NA	0.34

**Note**. $\text{GP}1707D07\text{ CL}/F\text{ retention ratio}=\frac{\;\text{GP}1707D07\;\text{CL}/F\;Period\;2}{\text{GP}1707D07\;\text{CL}/FPeriod\;1}$

$\text{GP}1707D07\text{ CL}/F\text{ reduction ratio}=\frac{\text{GP}1707D07\;\text{CL}/F\;Period\;1-\text{CL}/F\;Period\;2}{\text{GP}1707D07\;\text{CL}/F\;Period\;1}$

### 3.4 Safety

A total of 9 subjects (75.0%) experienced 22 treatment-emergent adverse events (TEAEs), all of which were of mild (CTCAE Grade 1) severity. All adverse events resolved/stabilized or recovered without medical intervention. No serious adverse events occurred in this study, and there were no adverse events leading to discontinuations, dose reduction, or withdraw. In the Suraxavir Marboxil (GP681) monotherapy stage (day 1 to day 21), 5 subjects (41.7%) experienced 9 adverse events. In the itraconazole monotherapy stage (day 22 to day 25), 4 subjects (33.3%) experienced 4 adverse events. In the Suraxavir Marboxil (GP681)-itraconazole co-administration stage (day 26 to day 36), 6 subjects (50.0%) experienced 9 adverse events (Table 6). The most common TEAE was investigation (6/12, 50.0%), followed by metabolism and nutrition disorders (4/12, 33.3%), and hepatobiliary disorders (1/12, 8.3%). Of the TEAEs above, 11 cases in 5 subjects were considered related to Suraxavir Marboxil (GP681) by the investigators. Among them, 4 subjects had 9 TEAEs during the Suraxavir Marboxil (GP681) monotherapy stage, and 2 subjects had 2 TEAEs during the Suraxavir Marboxil (GP681)-itraconazole co-administration stage (Table 7).

**TABLE 6:** Treatment-emergent adverse events.

System organ class Preferred term	GP681 monotherapy stage N = 12	Itraconazole monotherapy stage N = 12	GP681-itraconazole co-administration stage N = 12	Total N = 12
Overall total	5 (41.7)[9]	4 (33.3)[4]	6 (50.0)[9]	9 (75.0)[22]
Investigations	4 (33.3)[7]	2 (16.7)[2]	4 (33.3)[7]	6 (50.0)[16]
Alanine aminotransferase increased	1 (8.3)[1]	1 (8.3)[1]	2 (16.7)[2]	2 (16.7)[4]
Occult blood in urine	1 (8.3)[1]	1 (8.3)[1]	1 (8.3)[1]	2 (16.7)[3]
Gamma-glutamyltransferase increased	1 (8.3)[1]	0	1 (8.3)[1]	1 (8.3)[2]
Red blood cell increased	1 (8.3)[1]	0	1 (8.3)[1]	1 (8.3)[2]
Total bile acid increased	1 (8.3)[1]	0	0	1 (8.3)[1]
Lymphocyte count decreased	1 (8.3)[1]	0	0	1 (8.3)[1]
Basophil count increased	0	0	1 (8.3)[1]	1 (8.3)[1]
Hemoglobin decreased	1 (8.3)[1]	0	0	1 (8.3)[1]
White blood cell increased	0	0	1 (8.3)[1]	1 (8.3)[1]
Metabolism and nutrition disorders	2 (16.7)[2]	1 (8.3)	2 (16.7)[2]	4 (33.3)[5]
Hypophosphatemia	0	0	2 (16.7)[2]	2 (16.7)[2]
Hypertriglyceridemia	1 (8.3)[1])	1 (8.3)	0	1 (8.3)[2]
Hyperuricemia	1 (8.3)[1]	0	0	1 (8.3)[1]
Hepatobiliary disorders	0	1 (8.3)	0	1 (8.3)[1]
Hyperbilirubinemia	0	1 (8.3)	0	1 (8.3)[1]

Data are no. of subjects (percent of subjects) [no. of TEAEs].

**TABLE 7:** Treatment-emergent adverse events considered to be related to GP681.

System organ class Preferred term	GP681 monotherapy stage N = 12	GP681-itraconazole co-administration stage N = 12	Total N = 12
Overall total	4[9]	2[2]	5[11]
Investigations	3[7]	2[2]	3[9]
Alanine aminotransferase increased	1[1]	0	1[1]
Occult blood in urine	1[1]	1[1]	1[2]
Gamma-glutamyltransferase increased	1[1]	0	1[1]
Red blood cell increased	1[1]	1[1]	1[2]
Total bile acid increased	1[1]	0	1[1]
Lymphocyte count decreased	1[1]	0	1[1]
Hemoglobin decreased	1[1]	0	1[1]
Metabolism and nutrition disorders	2[2]	0	2[2]
Hypophosphatemia	1[1]	0	1[1]
Hypertriglyceridemia	1[1]	0	1[1]

Data are no. of subjects (percent of subjects) [no. of TEAEs].

## 4 Discussion

We chose a fixed-sequence design instead of the 2-sequence crossover design. The aim of such design was to maintain maximal CYP3A4 inhibition during GP1707D07 elimination, as well as to avoid the long washout period because of a delay in the washout of CYP3A4 inhibition and a potentially prolonged half-life of Suraxavir Marboxil (GP681) after concomitant dosing with itraconazole. The washout after Suraxavir Marboxil (GP681) administration spanned about 5 half-lives of GP1707D07 followed by a 3-day run-in (or lead-in) period prior to coadministration with Suraxavir Marboxil (GP681) to achieve adequately strong CYP3A4 inhibition. After the day of coadministration (day 26), itraconazole dosing continued for 14 days, covering 4–5 folds of estimated GP1707D07 half-life (Nolting et al., 1999; Clinical Drug Interaction Studies, 2024). However, the design had shortcomings, for we underestimated the half-life of GP1707D07 when used in combination with itraconazole, and designed the blood sampling time points in Period 2 to be the same as those in the Period 1. This design proved to be inadequate in ensuring sufficient blood sampling before the concentration of GP1707D07 decreased to a lower level. The concentration of GP1707D07 at the last blood sampling time point was still relatively high in 8 subjects, resulting in an extrapolated AUC to infinity (%AUC_extrap_) exceeding 20%. Therefore, these data were not included in the descriptive statistical analysis. Based on the available data, the exposure of GP1707D07 when co-administrated with itraconazole might have been underestimated.

As found in this study and in the single-dose escalation Phase I study, after oral administration, Suraxavir Marboxil (GP681) is rapidly converted into the active metabolite GP1707D07 in the body, leaving very low concentrations of the prodrug itself. The assessment of a DDI between Suraxavir Marboxil (GP681) and itraconazole was therefore performed on the basis of plasma concentration data of GP1707D07 instead of Suraxavir Marboxil (GP681).

Our present study showed that itraconazole, an inhibitor of CYP3A4, increased the GP1707D07 AUC_0- ∞_ by 2.5 folds and C_max_ by 1.4 folds of orally administered Suraxavir Marboxil (GP681) (Table 3, GeoMeans were based on the data of all 12 subjects). The differences in GP1707D07 concentration between Suraxavir Marboxil (GP681) monotherapy and Suraxavir Marboxil (GP681)-itraconazole co-administration had become evident since 36 h post-dose. Within 24 h post-dose, there were a total of 7 subjects whose GP1707D07 concentrations of some timepoints in Period 2 were not higher than in Period 1 (Subject ID: 301, 303, 305, 306, 308, 311, and 312 Supplementary Tables 4–7). The average ratio of GP1707D07 concentration at each time point (Period 2: Period 1) was around 1.7–2.0. However, from 36 h post-dose to the last quantifiable concentration, every timepoint had higher GP1707D07 concentration in Period 2 than in Period 1, and the ratio gradually increased from 2.0 to 4.4 (Supplementary Tables 4–7). When Suraxavir Marboxil (GP681) was co-administrated with itraconazole, the time to peak concentration of GP1707D07 only slightly increased (4.5 vs. 4.0 h), but the half-life was prolonged significantly (107.4 vs. 64.8 h), and the mean residence time in the body (MRT_0- ∞ _) was approximately doubled (170.5 vs. 87.2 h). Likewise, the C_max_ of GP1707D07 only exhibited a slight increase (22.0 vs. 16.0 ng/mL), but the systemic exposure was significantly increased (AUC_0- ∞_ 2,652.9 vs. 1,038.7 h*ng/mL, 2.5 folds). These findings suggested that inhibition of CYP3A4 might have a greater impact on the terminal elimination phase of GP1707D07 rather than the early stage of its metabolism.

The increase in C_max_ and AUC of GP1707D07 when Suraxavir Marboxil (GP681) was co-administrated with itraconazole did not lead to more adverse events. The incidence and severity of adverse events were similar during Period 1 and Period 2 (Tables 6, 7). It is notable that Subject 304 had 8.0 folds of C_max_ and 7.8 folds of AUC in Period 2 compared to Period 1, without any additional TEAEs during the co-administration period. These data confirmed the favorable safety profiles of Suraxavir Marboxil (GP681).

The expected therapeutic benefits of Suraxavir Marboxil (GP681) over currently approved treatments include faster resolution of influenza symptoms and a faster cessation of infectious virus shedding due to rapid reduction in virus titer. The estimated antiviral threshold for GP1707D07 was 6.87 ng/mL according to preclinical studies. In this study, when Suraxavir Marboxil (GP681) 20 mg was used in combination with itraconazole, GP1707D07 concentrations began to exceed the clinical effective concentration since 1 h post-dose, suggesting the reduction in dosage of Suraxavir Marboxil (GP681) during co-administration [a single dose of 40 mg is the current recommended regimen for Suraxavir Marboxil (GP681)] did not delay the onset of its antiviral effect.

There is no “golden standard” for precise dose adjustment based on DDI findings. The FDA guidance (Clinical Drug Interaction Studies, 2024) recommends a default no-effect boundary of 80%–125%, a change in systemic exposure measure within which is considered not clinically significant. Therefore, we interpret the 2.5-fold change of GP1707D07 systemic exposure during Suraxavir Marboxil (GP681)-itraconazole co-administration as a potential signal to warrant clinical action, such as dose adjustment or additional therapeutic monitoring. However, such no-effect boundary is generally considered very conservative for drugs that have wide safety margins. Given the specific features of Suraxavir Marboxil (GP681), the possibility that no dose adjustment is required when Suraxavir Marboxil (GP681) is used in combination with itraconazole cannot be excluded, for the following reasons: 1) Clinical trials of Suraxavir Marboxil (GP681) up to date have proved favorable safety profiles of Suraxavir Marboxil (GP681), with a wide therapeutic window. This DDI study showed no more safety concerns caused by increased systemic exposure of GP1707D07 during Suraxavir Marboxil (GP681)-itraconazole co-administration. 2) Co-administration with itraconazole only led to slight increase in the peak concentration of GP1707D07. The difference in plasma GP1707D07 concentration became evident after 36 h post-dose. CYP3A4 inhibitors significantly slow down the elimination of GP1707D07, but did not cause a surge in concentration. 3) The clinical dose regimen designed for Suraxavir Marboxil (GP681) is a 40 mg single oral dose. There are no concerns about drug accumulation or increased side effects caused by repeated doses of Suraxavir Marboxil (GP681).

This study also preliminarily reported the effects of CYP 1A2, CYP 2C19, and CYP 3A4 gene locus SNPs on the metabolism of GP1707D07 [either in Suraxavir Marboxil (GP681) monotherapy or in combination with itraconazole]. Due to the small sample size, some models (such as linear regression) were found to be unsuitable for effective analyses. For example, the stepwise regression model only identified the rs4646437 of CYP 3A4 as a significant independent variable relative to GP1707D07 CL/F, with a regression coefficient value of 14.855 (t = 3.007, *p* = 0.013 < 0.05, Table 8). Although it also indicates that the gene locus rs4646437 of CYP 3A4 has a significant positive impact on the metabolism of Suraxavir Marboxil (GP681), it may underestimate the role of other gene loci.

**TABLE 8:** Stepwise regression analysis results of cytochrome P450 SNPs in GP1707D07 metabolism (*n* = 12).

	Unstandardized coefficients		Standardized coefficients	*t*	*p*	Collinearity diagnosis	
	*B*	Std. Error	*Beta*			VIF	Tolerance
Constant	1.335	6.986	—	0.191	0.852	—	—
CYP 3A4_rs4646437	14.855	4.940	0.689	3.007	0.013*	1.000	1.000
*R* ^2^	0.475						
Adj *R* ^2^	0.422						
*F*	*F* (1,10) = 9.044, *p* = 0.013						
D-W value	2.241						

Note: Dependent Variable = GP1707D07 CL/F in Suraxavir Marboxil (GP681) monotherapy.

*p < 0.05.

The random forest algorithm employed in this study had considerable value for interpreting such results. Although the feature weight values predicted by this model might not quantitatively reflect the role of enzyme genotypes, the results indicated that the effects of cytochrome P450 enzymes followed the order of CYP 3A4 > CYP 1A2 > CYP 2C19. When CYP 3A4 was inhibited, CYP 1A2 played the key role in the metabolism of GP1707D07. Furthermore, based on the feature weight values of SNPs predicted by the model, combined with individual PK data and gene locus SNPs of the subjects, the impact of certain locus mutations on enzyme activity could be inferred, providing a better explanation for the inter-individual variability in PK data. Based on the present data, we hypothesized CYP 3A4 rs4646437 A>G, CYP 3A4 rs2246709 G>A, and CYP 2C19 rs12768009 A>G to be mutations that enhanced enzyme activity, while CYP1A2 rs762551 C>A weakened enzyme activity. These findings need further validation in research with larger samples.

## 5 Conclusion

Our present study showed that itraconazole, an inhibitor of CYP3A4, increased the GP1707D07 AUC_0- ∞_ by about 2.5 folds and C_max_ by about 1.4 folds of orally administered Suraxavir Marboxil (GP681). Differences in system exposure were more pronounced in the terminal elimination phase rather than in the early stage of GP1707D07 metabolism. Although the need for dosage adjustment in special population cannot be excluded, given the specific pharmacodynamic features and favorable safety profiles of Suraxavir Marboxil (GP681), we consider the PK change of GP1707D07 during itraconazole co-administration inadequate to warrant clinical action. Given the limitations of the study design, the clinical effect of CYP 3A4 inhibition on Suraxavir Marboxil remains inconclusive. Further studies are needed to clarify the impact of CYP 3A4 inhibitors on Suraxavir Marboxil. Gene polymorphism did not pose a safety concern for Suraxavir Marboxil (GP681). By estimating feature values of specific SNPs, the random forest algorithm contributes to the understanding of pharmacogenetic variants involved in GP1707D07 metabolism. It may be a potent tool for assessing of the impact of gene polymorphism on PK profiles in small clinical samples.

Suraxavir Marboxil (GP681) is a promising novel antiviral drug, with more clinical studies currently underway. The clinical impact of Suraxavir Marboxil (GP681)-itraconazole DDI will be more explicit when an exposure-response relationship is clearly defined in the future.

## Data Availability

The original contributions presented in the study are publicly available. This data can be found here: https://www.ncbi.nlm.nih.gov/SNP/snp_viewBatch.cgi?sbid=1063702.

## References

[B1] Becker T. Rousseau A. J. Geubbelmans M. Burzykowski T. Valkenborg D. (2023). Decision trees and random forests. Am. J. Orthod. Dentofac. Orthop. 164 (6), 894–897. 10.1016/j.ajodo.2023.09.011 38008491

[B2] Breiman L. (2001). Random forests. Mach. Learn. 45, 5–32. 10.1023/a:1010933404324

[B3] Caceres C. J. Seibert B. Cargnin Faccin F. Cardenas-Garcia S. Rajao D. S. Perez D. R. (2022). Influenza antivirals and animal models. FEBS Open Bio 12 (6), 1142–1165. 10.1002/2211-5463.13416 PMC9157400 35451200

[B4] Clinical Drug Interaction Studies (2024). Clinical drug interaction studies — cytochrome P450 enzyme- and transporter-mediated drug interactions guidance for industry. Available at: https://www.fda.gov/regulatory-information/search-fda-guidance-documents/clinical-drug-interaction-studies-cytochrome-p450-enzyme-and-transporter-mediated-drug-interactions (Accessed August 2. 2024).

[B5] De Clercq E. (2006). Antiviral agents active against influenza A viruses. Nat. Rev. Drug Discov. 5 (12), 1015–1025. 10.1038/nrd2175 17139286 PMC7097821

[B6] Dias A. Bouvier D. Crépin T. McCarthy A. A. Hart D. J. Baudin F. (2009). The cap-snatching endonuclease of influenza virus polymerase resides in the PA subunit. Nature 458 (7240), 914–918. 10.1038/nature07745 19194459

[B7] Heykants J. Van Peer A. Van de Velde V. Van Rooy P. Meuldermans W. Lavrijsen K. (1989). The clinical pharmacokinetics of itraconazole: an overview. Mycoses 32 (Suppl. 1), 67–87. 10.1111/j.1439-0507.1989.tb02296.x 2561187

[B8] Ho T. K. (1995). “Random decision forests,” in Proceedings of 3rd International Conference on Document Analysis and Recognition (Piscataway, NJ: IEEE), 278–282.

[B9] Hu J. Szymczak S. (2023). A review on longitudinal data analysis with random forest. Brief. Bioinform 24 (2), bbad002. 10.1093/bib/bbad002 36653905 PMC10025446

[B10] Huynh-Thu V. A. Geurts P. (2019). Unsupervised gene network inference with decision trees and random forests. Methods Mol. Biol. 1883, 195–215. 10.1007/978-1-4939-8882-2_8 30547401

[B11] Ich M12 on Drug Interaction Studies (2024). ICH M12 on drug interaction studies - scientific guideline. Available at: https://www.ema.europa.eu/en/ich-m12-drug-interaction-studies-scientific-guideline (Accessed July 4. 2024).

[B12] Ikematsu H. Hayden F. G. Kawaguchi K. Kinoshita M. de Jong M. D. Lee N. (2020). Baloxavir marboxil for prophylaxis against influenza in household contacts. N. Engl. J. Med. 383 (4), 309–320. 10.1056/NEJMoa1915341 32640124

[B13] Jin Y. Lan A. Dai Y. Jiang L. Liu S. (2023). Development and testing of a random forest-based machine learning model for predicting events among breast cancer patients with a poor response to neoadjuvant chemotherapy. Eur. J. Med. Res. 28 (1), 394. 10.1186/s40001-023-01361-7 37777809 PMC10543332

[B14] Jones J. C. Yen H. L. Adams P. Armstrong K. Govorkova E. A. (2023). Influenza antivirals and their role in pandemic preparedness. Antivir. Res. 210, 105499. 10.1016/j.antiviral.2022.105499 36567025 PMC9852030

[B15] Ke A. B. Zamek-Gliszczynski M. J. Higgins J. W. Hall S. D. (2014). Itraconazole and clarithromycin as ketoconazole alternatives for clinical CYP3A inhibition studies. Clin. Pharmacol. Ther. 95 (5), 473–476. 10.1038/clpt.2014.41 24747234

[B16] Krammer F. Smith G. J. D. Fouchier R. A. M. Peiris M. Kedzierska K. Doherty P. C. (2018). Influenza. Nat. Rev. Dis. Prim. 4 (1), 3. 10.1038/s41572-018-0002-y 29955068 PMC7097467

[B17] Kuo Y. C. Lai C. C. Wang Y. H. Chen C. H. Wang C. Y. (2021). Clinical efficacy and safety of baloxavir marboxil in the treatment of influenza: a systematic review and meta-analysis of randomized controlled trials. J. Microbiol. Immunol. Infect. 54 (5), 865–875. 10.1016/j.jmii.2021.04.002 34020891

[B18] Liu J. W. Lin S. H. Wang L. C. Chiu H. Y. Lee J. A. (2021). Comparison of antiviral agents for seasonal influenza outcomes in healthy adults and children: a systematic review and network meta-analysis. JAMA Netw. Open 4 (10), e2119151. 10.1001/jamanetworkopen.2021.19151 34387680 PMC8363918

[B19] Liu L. Bello A. Dresser M. J. Heald D. Komjathy S. F. O'Mara E. (2016). Best practices for the use of itraconazole as a replacement for ketoconazole in drug-drug interaction studies. J. Clin. Pharmacol. 56 (2), 143–151. 10.1002/jcph.562 26044116

[B20] Nolting S. K. Gupta A. Doncker P. D. Jacko M. L. Moskovitz B. L. (1999). Continuous itraconazole treatment for onychomycosis and dermatomycosis: an overview of safety. Eur. J. Dermatol 9 (7), 540–543. 10523732

[B21] Noshi T. Kitano M. Taniguchi K. Yamamoto A. Omoto S. Baba K. (2018). *In vitro* characterization of baloxavir acid, a first-in-class cap-dependent endonuclease inhibitor of the influenza virus polymerase PA subunit. Antivir. Res. 160, 109–117. 10.1016/j.antiviral.2018.10.008 30316915

[B22] O'Hanlon R. Shaw M. L. (2019). Baloxavir marboxil: the new influenza drug on the market. Curr. Opin. Virol. 35, 14–18. 10.1016/j.coviro.2019.01.006 30852344

[B23] Omoto S. Speranzini V. Hashimoto T. Noshi T. Yamaguchi H. Kawai M. (2018). Characterization of influenza virus variants induced by treatment with the endonuclease inhibitor baloxavir marboxil. Sci. Rep. 8 (1), 9633. 10.1038/s41598-018-27890-4 29941893 PMC6018108

[B24] Piérard G. E. Arrese J. E. Piérard-Franchimont C. (2000). Itraconazole. Expert Opin. Pharmacother. 1 (2), 287–304. 10.1517/14656566.1.2.287 11249550

[B25] Shi G. Liu G. Gao Q. Zhang S. Wang Q. Wu L. (2023). A random forest algorithm-based prediction model for moderate to severe acute postoperative pain after orthopedic surgery under general anesthesia. BMC Anesthesiol. 23 (1), 361. 10.1186/s12871-023-02328-1 37932714 PMC10626723

[B26] Takashita E. Morita H. Ogawa R. Nakamura K. Fujisaki S. Shirakura M. (2018). Susceptibility of influenza viruses to the novel cap-dependent endonuclease inhibitor baloxavir marboxil. Front. Microbiol. 9, 3026. 10.3389/fmicb.2018.03026 30574137 PMC6291754

[B27] Technical Guidelines for Drug Interaction Studies (2024).Technical guidelines for drug interaction studies. Available at: https://www.cqn.com.cn/ms/att/2021-02/09/288fc41f-730e-47df-8ad2-a025ee71ee9d.pdf (Accessed July 4. 2024).

[B28] Templeton I. E. Thummel K. E. Kharasch E. D. Kunze K. L. Hoffer C. Nelson W. L. (2008). Contribution of itraconazole metabolites to inhibition of CYP3A4 *in vivo* . Clin. Pharmacol. Ther. 83 (1), 77–85. 10.1038/sj.clpt.6100230 17495874 PMC3488349

[B29] Tian L. Wu W. Yu T. (2023). Graph random forest: a graph embedded algorithm for identifying highly connected important features. Biomolecules 13 (7), 1153. 10.3390/biom13071153 37509188 PMC10377046

[B30] Zhao Y. Gao Y. Guyatt G. Uyeki T. M. Liu P. Liu M. (2024). Antivirals for post-exposure prophylaxis of influenza: a systematic review and network meta-analysis. Lancet 404 (10454), 764–772. 10.1016/S0140-6736(24)01357-6 39181596 PMC11369964

