# Supplementary material for: Evaluation of drug-drug interaction between Suraxavir Marboxil (GP681) and itraconazole, and assessment of the impact of gene polymorphism
Source: Front Pharmacol. 2025 Apr 11;15:1505557. doi: 10.3389/fphar.2024.1505557 (PMC12022903; doi:10.3389/fphar.2024.1505557)
Supplement: Supplementary file 1 [file DataSheet1.docx]

**Evaluation of Drug-Drug Interaction between Suraxavir Marboxil (GP681) and Itraconazole, and Assessment of the Impact of Gene Polymorphism.**

Mai Han, Gang Cui, Yan Zhao, Xianbo Zuo, Xiaoxue Wang, Xin Zhang, Na Mi, Jiangli Jin, Chunyan Xiao, Jing Wang, Wei Wu, Yajuan Li, and Jintong Li

**SUPPLEMENTARY MATERIAL**

1. **Supplementary methods**

**1.1 Supplementary Table 1. Summary of Validation Result**

| Contents of the Validation | Results |
| --- | --- |
| Stock Solution Bench-top Stability of Analyte | The analyte was stable at RT white light and yellow light for 23 hours in MeOH solution |
| Spike Solution Bench-top Stability of Analyte (ULOQ) | The analyte was stable at RT white light for 23 hours in MeOH/H2O (1:1, v/v) solution |
| Spike Solution Bench-top Stability of Analyte (LLOQ) | The analyte was stable at RT white light for 23 hours in MeOH/H2O (1:1, v/v) solution |
| Stock Solution Bench-top Stability of IS | The IS was stable at RT white light and yellow light for 23 hours in MeOH solution |
| Spike solution Bench-top stability of IS | The IS was stable at RT white light for 23 hours in MeOH/H2O (1:1, v/v) solution |
| Stock Solution Long-term Stability of Analyte | The analyte was stable at -20°C for 48 days in MeOH solution |
| Spike Solution Long-term Stability of Analyte (ULOQ) | The analyte was stable at -20°C for 48 days in MeOH/H2O (1:1, v/v) solution |
| Spike Solution Long-term Stability of Analyte (LLOQ) | The analyte was stable at -20°C for 48 days in MeOH/H2O (1:1, v/v) solution |
| Stock Solution Long-term Stability of IS | The IS was stable at -20°C for 48 days in MeOH solution |
| Spike Solution Long-term Stability of IS | The IS was stable at -20°C for 48 days in MeOH/H2O (1:1, v/v) solution |
| Blood Stability | The analyte was stable for 2 hours in Ice-water bath in whole blood |
| Bench-Top Stability in Matrix | The analyte was stable for 25 hours in Ice-water bath in human plasma, the analyte was stable for 34 hours at RT in human plasma |
| Processed Sample Stability | The analyte was stable for 101 hours in the autosampler 4°C |
| Freeze/Thaw Stability | The analyte was stable for 5 freeze/thaw cycles at -80°C/Ice-water bath and -20°C/Ice-water bath in human plasma |
| Long-term Stability in Matrix | The analyte was stable for 49 days at -20°C and was stable for 522 days at -80°C in human plasma |
| Long-term Stability in Matrix (contain Oseltamivir,Oseltamivir carboxylic acid and Itraconazole) | The analyte was stable for 127 days at -20°C and -80°C in human plasma which the matrix containing the potentially interfering drugs (Oseltamivir, Oseltamivir carboxylic acid and Itraconazole) |
| Reinjection Reproducibility Stability | The analyte was stable for 109 hours in the autosampler 4°C |

**1.2 Supplementary Table 2. Ratio of Integrated Area of Test Sample to Internal Standard Compound**

| Bio-matrix Batch Number | Concentration: 40 ng/mL | | |
| --- | --- | --- | --- |
|  | GP1707D06 Area in Bio-matrix | GP681 Area in Bio-matrix | GP1707D07 Area in Bio-matrix |
| SY-KBX-001 | 912580 | 1255770 | 1372291 |
| SY-KBX-002 | 904460 | 1310175 | 1414243 |
| SY-KBX-003 | 924350 | 1294828 | 1352411 |
| SY-KBX-004 | 901487 | 1273922 | 1365265 |
| SY-KBX-005 | 905852 | 1271730 | 1399255 |
| SY-KBX-006 | 902788 | 1292141 | 1370310 |
| Mean | 908586 | 1283094 | 1378963 |
| Ratio of Peak Area of Analyte to Internal Standard |  | 1.41 | 1.52 |

**1.3 Pharmacokinetic Sample Collection and Processing:** Pharmacokinetic Analysis: Approximately 4 mL of blood is collected each time into a tube containing sodium fluoride/potassium oxalate as an anticoagulant. The tube is then placed upright in an ice-water bath. The plasma samples are separated by centrifugation for the detection of GP681/GP1707D07 blood drug concentrations. Both sample collection and processing are conducted under white light conditions. After collection, the whole blood samples are placed in a low-temperature centrifuge set at 4°C (allowable range: 2°C to 8°C, 1800g, with a centrifugal force of 1500g for samples used for GP681/GP1707D07 blood drug concentration detection) within 1 hour of collection and centrifuged for 10 minutes, followed by the separation of plasma samples. For each centrifuged plasma sample, 0.8 mL of plasma is transferred into a detection storage tube, with the aliquotting process performed in an ice-water bath. An LC-MS/MS method is used for the determination of GP681 and GP1707D07 in NaF/KoX Human Plasma, using GP1707D06 as the internal standard (IS). GP681, GP1707D07, and GP1707D06 are extracted by protein precipitation using 0.1% formic acid in acetonitrile (ACN) from human NaF/KoX plasma.

**1.4 Genetic Polymorphism Sample Collection and Processing:** Peripheral venous blood (4 mL) is collected from the subject before drug administration on Day 1 into a tube containing EDTA-K2 as an anticoagulant and placed upright at room temperature. 0.8 mL of whole blood is transferred into a detection storage tube for genetic polymorphism analysis.

**1.5 Supplementary Table 3. Retention Times of Analyte and Internal Standard**

| Compound | Retention Time (minutes) |
| --- | --- |
| Analyte (GP681) | 1.1 |
| Analyte (GP1707D07) | 1.0 |
| Internal Standard (GP1707D06) | 1.5 |

**1.6 Supplementary Figure 1: Representative chromatogram of the GP681 and IS（GP1707D06）at LLOQ**


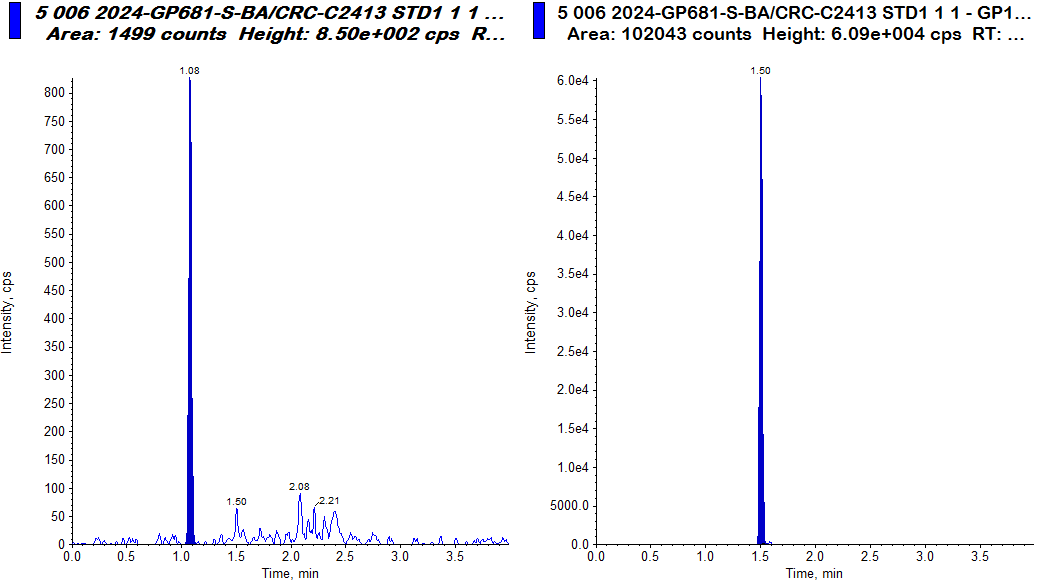


**1.7 Supplementary Figure 2: Representative chromatogram of the GP1707D07 and IS（GP1707D06）at LLOQ**


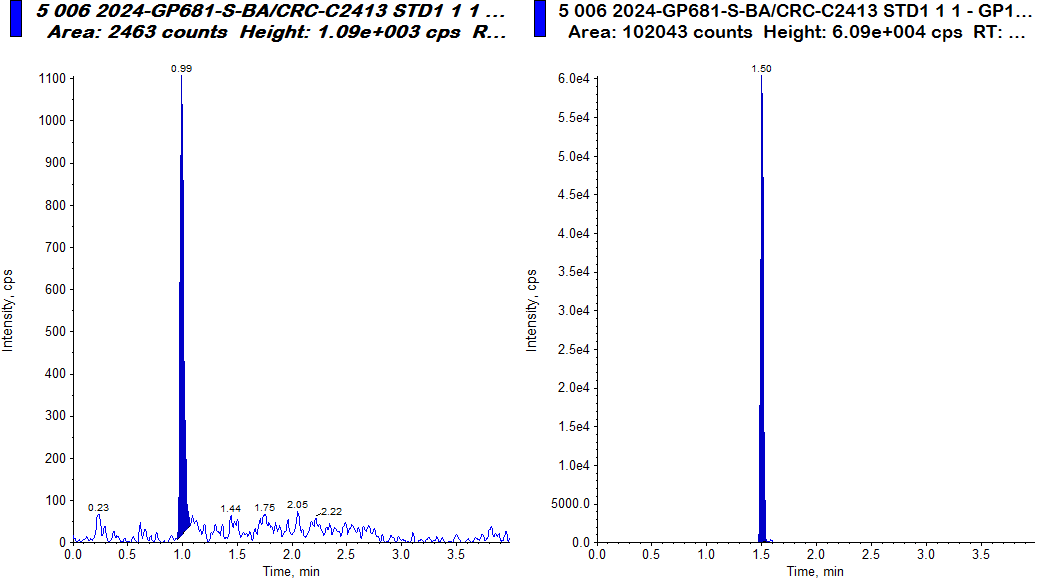


1. **Supplementary results**

| **2.1 Supplementary Table 4. Pharmacokinetic Parameters -** **Pharmacokinetic Parameter Set for GP1707D07** | | | | | | | | | | | |
| --- | --- | --- | --- | --- | --- | --- | --- | --- | --- | --- | --- |
|  | Period 1: Suraxavir Marboxil (GP681) monotherapy | | | | | | | | | | |
| Subject ID | C_max_ (ng/mL) | AUC_0-t_ (h*ng/mL) | AUC_0-∞_ (h*ng/mL) | T_max_ (h) | t_1/2_ (h) | λz (h-1) | CL/F (L/h) | V_z_/F (L) | AUC__%Extrap_ (%) | MRT_0-t_ (h) | MRT_0-∞_ (h) |
| 301 | 16.03 | 759.35 | 781.02 | 4 | 48.78 | 0.014 | 25.61 | 1801.98 | 2.78 | 57.28 | 64.34 |
| 302 | 20.468 | 1247.30 | 1310.49 | 6 | 53.74 | 0.013 | 15.26 | 1183.17 | 4.82 | 63.69 | 75.97 |
| 303 | 30.14 | 1364.54 | 1409.63 | 3 | 48.69 | 0.014 | 14.19 | 996.59 | 3.20 | 56.39 | 64.54 |
| 304 | 3.382 | 354.04 | 407.77 | 6 | 81.51 | 0.009 | 49.05 | 5767.31 | 13.18 | 80.89 | 117.49 |
| 305 | 11.179 | 739.81 | 814.16 | 4 | 71.18 | 0.010 | 24.57 | 2522.77 | 9.13 | 69.22 | 94.32 |
| 306 | 44.439 | 1770.18 | 1874.52 | 3 | 55.68 | 0.012 | 10.67 | 857.04 | 5.57 | 61.44 | 75.90 |
| 307 | 17.988 | 1069.77 | 1171.14 | 6 | 70.05 | 0.010 | 17.08 | 1725.92 | 8.66 | 71.79 | 95.15 |
| 308 | 26.933 | 1199.27 | 1273.75 | 2 | 61.90 | 0.011 | 15.70 | 1402.22 | 5.85 | 62.61 | 78.26 |
| 309 | 7.24 | 602.25 | 689.41 | 2 | 72.43 | 0.010 | 29.01 | 3031.63 | 12.64 | 74.73 | 108.96 |
| 310 | 15.539 | 1158.79 | 1290.37 | 5 | 72.44 | 0.010 | 15.50 | 1619.87 | 10.20 | 74.78 | 102.37 |
| 311 | 12.018 | 728.18 | 801.51 | 5 | 68.41 | 0.010 | 24.95 | 2462.69 | 9.15 | 69.62 | 94.31 |
| 312 | 25.999 | 1487.97 | 1650.91 | 2 | 84.86 | 0.008 | 12.11 | 1483.10 | 9.87 | 63.68 | 93.23 |
| n | 12 | 12 | 12 | 12 | 12 | 12 | 12 | 12 | 12 | 12 | 12 |
| Mean | 19.2796 | 1040.120 | 1122.891 | 4.0 | 65.805 | 0.0109 | 21.141 | 2071.190 | 7.919 | 67.176 | 88.737 |
| SD | 11.2748 | 410.1866 | 429.4908 | 1.60 | 12.0517 | 0.0021 | 10.6123 | 1333.0493 | 3.4463 | 7.5498 | 16.9581 |
| CV% | 58.48 | 39.44 | 38.25 | 39.89 | 18.31 | 19.25 | 50.20 | 64.36 | 43.52 | 11.24 | 19.11 |
| Median | 17.0090 | 1114.281 | 1222.445 | 4.0 | 69.231 | 0.0100 | 16.390 | 1672.897 | 8.894 | 66.454 | 93.771 |
| Q1 | 11.5985 | 733.993 | 791.267 | 2.5 | 54.708 | 0.0096 | 14.725 | 1292.694 | 5.194 | 62.024 | 75.937 |
| Q3 | 26.4660 | 1305.919 | 1360.058 | 5.5 | 72.438 | 0.0127 | 25.280 | 2492.730 | 10.034 | 73.262 | 98.759 |

| Min | 3.382 | 354.04 | 407.77 | 2 | 48.69 | 0.008 | 10.67 | 857.04 | 2.78 | 56.39 | 64.34 |
| --- | --- | --- | --- | --- | --- | --- | --- | --- | --- | --- | --- |
| Max | 44.439 | 1770.18 | 1874.52 | 6 | 84.86 | 0.014 | 49.05 | 5767.31 | 13.18 | 80.89 | 117.49 |
| Geo Mean | 16.0070 | 955.800 | 1038.671 | 3.7 | 64.764 | 0.0107 | 19.255 | 1799.125 | 7.117 | 66.790 | 87.218 |
| Geo CV% | 78.5016 | 47.8377 | 45.4270 | 46.08 | 19.0118 | 19.0118 | 45.4270 | 56.3019 | 54.7780 | 11.2458 | 19.7659 |
|  | | | | | | | | | | | |

|  | Period 2: Suraxavir Marboxil (GP681)-Itraconazole co-administration | | | | | | | | | | |  |
| --- | --- | --- | --- | --- | --- | --- | --- | --- | --- | --- | --- | --- |
| Subject ID | C_max_ (ng/mL) | AUC_0-t_ (h*ng/mL) | AUC_0-∞_ (h*ng/mL) | T_max_ (h) | t_1/2_ (h) | λz (h-1) | CL/F (L/h) | V_z_/F (L) | AUC__%Extrap_ (%) | MRT_0-t_ (h) | MRT_0-∞_ (h) |  |
| 301 | 20.426 | 1661.62 | 1943.92 | 5 | 91.31 | 0.008 | 10.29 | 1355.33 | 14.52 | 84.46 | 129.66 |  |
| 302 | 33.588 | 2484.54 | 3038.12 | 3 | 115.86 | 0.006 | 6.58 | 1100.32 | 18.22 | 87.34 | 149.99 |  |
| 303 | 31.705 | 2488.27 | 3010.01 | 4 | 115.17 | 0.006 | 6.64 | 1104.05 | 17.33 | 83.83 | 143.87 |  |
| 304 | 26.988 | 2757.79 | 3496.61 | 4 | 123.85 | 0.006 | 5.72 | 1021.99 | 21.13 | 94.91 | 168.40 |  |
| 305 | 14.549 | 1586.83 | 2164.09 | 6 | 154.25 | 0.004 | 9.24 | 2056.61 | 26.67 | 93.53 | 198.32 |  |
| 306 | 26.492 | 2524.54 | 3287.23 | 5 | 134.65 | 0.005 | 6.08 | 1181.94 | 23.20 | 94.49 | 178.88 |  |
| 307 | 30.409 | 2991.14 | 4071.31 | 6 | 147.04 | 0.005 | 4.91 | 1042.08 | 26.53 | 95.36 | 196.38 |  |
| 308 | 21.586 | 2032.39 | 2952.73 | 4 | 172.37 | 0.004 | 6.77 | 1684.38 | 31.17 | 94.95 | 225.05 |  |
| 309 | 13.021 | 1353.85 | 1815.81 | 5 | 120.20 | 0.006 | 11.01 | 1909.99 | 25.44 | 95.71 | 182.53 |  |
| 310 | 20.877 | 2127.20 | 2809.94 | 3 | 119.29 | 0.006 | 7.12 | 1224.96 | 24.30 | 95.46 | 178.20 |  |
| 311 | 9.944 | 1044.79 | 1322.58 | 5 | 123.51 | 0.006 | 15.12 | 2694.46 | 21.00 | 92.09 | 165.60 |  |
| 312 | 32.652 | 2764.96 | 3386.84 | 4 | 109.15 | 0.006 | 5.91 | 929.93 | 18.36 | 90.98 | 151.62 |  |
|  | | | | | | | | | | | |  |
| n | 12 | 12 | 12 | 12 | 12 | 12 | 12 | 12 | 12 | 12 | 12 |  |
| Mean | 23.5198 | 2151.494 | 2774.932 | 4.5 | 127.221 | 0.0056 | 7.951 | 1442.169 | 22.324 | 91.926 | 172.374 |  |
| SD | 8.0644 | 621.3664 | 802.7575 | 1.00 | 21.8352 | 0.0009 | 2.9462 | 537.3273 | 4.7742 | 4.3589 | 26.6878 |  |
| CV% | 34.29 | 28.88 | 28.93 | 22.22 | 17.16 | 16.73 | 37.06 | 37.26 | 21.39 | 4.74 | 15.48 |  |
| Median | 24.0390 | 2305.872 | 2981.370 | 4.5 | 121.852 | 0.0057 | 6.709 | 1203.452 | 22.166 | 94.010 | 173.300 |  |
| Q1 | 17.4875 | 1624.228 | 2054.007 | 4.0 | 115.515 | 0.0049 | 5.995 | 1071.198 | 18.291 | 89.159 | 150.805 |  |
| Q3 | 31.0570 | 2641.166 | 3337.031 | 5.0 | 140.847 | 0.0060 | 9.765 | 1797.182 | 25.986 | 95.156 | 189.454 |  |
| Min | 9.944 | 1044.79 | 1322.58 | 3 | 91.31 | 0.004 | 4.91 | 929.93 | 14.52 | 83.83 | 129.66 |  |
| Max | 33.588 | 2991.14 | 4071.31 | 6 | 172.37 | 0.008 | 15.12 | 2694.46 | 31.17 | 95.71 | 225.05 |  |

| Geo Mean | 22.0359 | 2057.031 | 2652.846 | 4.4 | 125.561 | 0.0055 | 7.539 | 1365.672 | 21.847 | 91.828 | 170.494 |
| --- | --- | --- | --- | --- | --- | --- | --- | --- | --- | --- | --- |
| Geo CV% | 41.3306 | 33.5834 | 33.6634 | 23.42 | 16.9767 | 16.9767 | 33.6634 | 34.1805 | 22.1772 | 4.8557 | 15.5799 |
|  | | | | | | | | | | | |

**2.2 Supplementary Table 5** Plasma GP1707D07 concentrations-time profiles of individual subjects in GP681 monotherapy stage.

| Period 1  GP681 monotherapy  （N=12） | GP1707D07 concentration (ng/mL) | | | | | | | | | | | | | | | | |
| --- | --- | --- | --- | --- | --- | --- | --- | --- | --- | --- | --- | --- | --- | --- | --- | --- | --- |
|  | 0h | 0.5h | 1h | 2h | 3h | 4h | 5h | 6h | 8h | 12h | 24h | 36h | 48h | 72h | 120h | 168h | 264h |
| Subject ID | D1 C1 | D1 C2 | D1 C3 | D1 C4 | D1 C5 | D1 C6 | D1 C7 | D1 C8 | D1 C9 | D1 C10 | D1 C11 | D1 C12 | D1 C13 | D1 C14 | D1 C15 | D1 C16 | D1 C17 |
| 301 | BQL | 3.4 | 7.8 | 11.6 | 12.9 | 16.0 | 12.5 | 14.3 | 11.5 | 11.0 | 8.1 | 7.0 | 4.9 | 3.6 | 1.8 | 1.0 | 0.3 |
| 302 | BQL | 5.0 | 6.6 | 9.1 | 20.3 | 15.8 | 14.6 | 20.5 | 16.4 | 12.7 | 14.3 | 10.7 | 9.4 | 6.7 | 3.3 | 1.7 | 0.8 |
| 303 | BQL | 17.1 | 21.9 | 25.1 | 30.1 | 27.7 | 24.4 | 23.1 | 21.7 | 15.4 | 16.5 | 11.0 | 9.3 | 6.8 | 3.4 | 1.4 | 0.6 |
| 304 | BQL | 2.6 | 3.0 | 2.8 | 2.9 | 3.1 | 3.3 | 3.4 | 3.2 | 3.0 | 3.2 | 2.5 | 2.2 | 1.7 | 1.4 | 0.8 | 0.5 |
| 305 | BQL | 4.5 | 8.1 | 9.1 | 10.7 | 11.2 | 10.0 | 10.1 | 9.3 | 8.2 | 7.1 | 6.3 | 4.5 | 3.8 | 2.2 | 1.3 | 0.7 |
| 306 | BQL | 17.9 | 23.5 | 33.1 | 44.4 | 36.1 | 30.8 | 30.7 | 24.5 | 21.5 | 17.5 | 14.8 | 12.3 | 7.8 | 4.8 | 2.4 | 1.3 |
| 307 | BQL | 0.6 | 1.8 | 4.5 | 11.9 | 13.4 | 14.9 | 18.0 | 14.2 | 12.6 | 10.3 | 8.1 | 6.9 | 5.1 | 3.5 | 2.0 | 1.0 |
| 308 | BQL | 10.8 | 16.5 | 26.9 | 25.1 | 24.0 | 20.4 | 19.4 | 17.9 | 14.5 | 12.6 | 10.5 | 7.4 | 4.9 | 3.2 | 1.9 | 0.8 |
| 309 | BQL | 2.8 | 6.2 | 7.2 | 6.4 | 5.7 | 7.0 | 7.0 | 6.9 | 6.6 | 5.4 | 4.4 | 3.9 | 3.2 | 1.7 | 1.1 | 0.8 |
| 310 | BQL | 8.3 | 10.1 | 12.6 | 14.2 | 15.4 | 15.5 | 15.2 | 13.5 | 11.1 | 10.1 | 8.8 | 6.7 | 6.0 | 4.0 | 2.2 | 1.3 |
| 311 | BQL | 1.4 | 5.6 | 9.4 | 10.4 | 11.0 | 12.0 | 11.0 | 9.5 | 8.8 | 6.3 | 5.4 | 5.1 | 3.6 | 2.1 | 1.3 | 0.7 |
| 312 | BQL | 11.4 | 18.8 | 26.0 | 25.2 | 23.8 | 24.9 | 23.3 | 22.5 | 20.0 | 14.7 | 11.8 | 9.0 | 7.6 | 3.6 | 2.2 | 1.3 |
| Mean | 0.0 | 7.2 | 10.8 | 14.8 | 17.9 | 16.9 | 15.9 | 16.3 | 14.3 | 12.1 | 10.5 | 8.4 | 6.8 | 5.1 | 2.9 | 1.6 | 0.9 |
| SD | 0.0 | 6.0 | 7.4 | 10.1 | 11.7 | 9.4 | 8.0 | 7.7 | 6.6 | 5.3 | 4.6 | 3.5 | 2.8 | 1.9 | 1.0 | 0.5 | 0.3 |
| CV% | NA | 83.2 | 68.5 | 68.6 | 65.4 | 55.8 | 50.1 | 47.3 | 46.3 | 43.7 | 44.1 | 41.4 | 41.9 | 38.4 | 35.9 | 34.1 | 37.8 |
| Geo Mean | 1.0 | 4.8 | 8.4 | 11.6 | 14.4 | 14.2 | 13.7 | 14.2 | 12.5 | 10.9 | 9.4 | 7.7 | 6.2 | 4.7 | 2.7 | 1.5 | 0.8 |
| Geo CV% | 0.0 | 136.7 | 93.2 | 88.4 | 85.3 | 77.2 | 67.4 | 67.3 | 63.3 | 57.0 | 55.2 | 52.5 | 49.8 | 47.6 | 39.9 | 38.3 | 45.2 |

**2.3 Supplementary Table 6** Plasma GP1707D07 concentrations-time profiles of individual subjects in GP681-itraconazole co-administration stage.

| Period 2 GP681-itraconazole co-administration | GP1707D07 concentration (ng/mL) | | | | | | | | | | | | | | | | |
| --- | --- | --- | --- | --- | --- | --- | --- | --- | --- | --- | --- | --- | --- | --- | --- | --- | --- |
|  | D26 C18 | D26 C19 | D26 C20 | D26 C21 | D26 C22 | D26 C23 | D26 C24 | D26 C25 | D26 C26 | D26 C27 | D26 C28 | D26 C29 | D26 C30 | D26 C31 | D26 C32 | D26 C33 | D26 C34 |
| Subject ID | 0h | 0.5h | 1h | 2h | 3h | 4h | 5h | 6h | 8h | 12h | 24h | 36h | 48h | 72h | 120h | 168h | 264h |
| 301 | BQL | 3.5 | 6.8 | 13.4 | 18.3 | 19.9 | 20.4 | 19.5 | 18.6 | 16.8 | 12.3 | 11.8 | 10.1 | 7.5 | 6.0 | 3.3 | 2.1 |
| 302 | BQL | 11.4 | 16.5 | 29.1 | 33.6 | 30.2 | 29.3 | 28.8 | 23.8 | 21.6 | 18.7 | 17.6 | 13.3 | 11.8 | 7.9 | 6.0 | 3.3 |
| 303 | BQL | 10.9 | 14.6 | 28.7 | 31.3 | 31.7 | 29.9 | 30.1 | 26.2 | 22.1 | 19.9 | 18.2 | 15.0 | 11.9 | 7.6 | 5.4 | 3.1 |
| 304 | BQL | 12.7 | 15.7 | 21.1 | 25.8 | 27.0 | 25.8 | 24.6 | 24.4 | 19.2 | 19.6 | 17.3 | 16.0 | 12.2 | 9.4 | 7.8 | 4.1 |
| 305 | BQL | 1.7 | 5.8 | 10.7 | 12.8 | 13.3 | 13.8 | 14.5 | 13.2 | 12.3 | 11.4 | 11.1 | 9.6 | 7.7 | 4.9 | 4.1 | 2.6 |
| 306 | BQL | 0.7 | 2.3 | 5.8 | 7.8 | 13.8 | 26.5 | 26.3 | 23.3 | 21.2 | 17.6 | 15.6 | 14.1 | 13.4 | 8.2 | 6.6 | 3.9 |
| 307 | BQL | 4.4 | 9.2 | 18.9 | 24.7 | 27.1 | 27.1 | 30.4 | 25.8 | 24.6 | 22.7 | 18.3 | 17.7 | 12.9 | 9.6 | 8.2 | 5.1 |
| 308 | BQL | 6.4 | 11.2 | 13.9 | 18.7 | 21.6 | 20.0 | 18.8 | 19.5 | 15.5 | 14.3 | 13.0 | 12.2 | 9.0 | 6.7 | 5.1 | 3.7 |
| 309 | BQL | 5.1 | 9.9 | 11.2 | 10.9 | 10.6 | 13.0 | 12.7 | 11.8 | 10.3 | 9.6 | 8.3 | 8.6 | 6.2 | 4.3 | 3.3 | 2.7 |
| 310 | BQL | 9.7 | 13.6 | 16.5 | 20.9 | 18.0 | 20.8 | 19.4 | 19.6 | 16.0 | 14.8 | 13.2 | 12.5 | 9.6 | 7.4 | 5.2 | 4.0 |
| 311 | BQL | 0.7 | 1.8 | 7.7 | 8.8 | 9.7 | 9.9 | 9.3 | 8.6 | 8.2 | 7.6 | 6.9 | 6.2 | 5.2 | 3.5 | 2.6 | 1.6 |
| 312 | BQL | 9.1 | 17.7 | 26.9 | 23.4 | 32.7 | 27.5 | 25.4 | 25.9 | 23.2 | 18.5 | 18.6 | 15.2 | 13.6 | 9.6 | 6.8 | 3.9 |
| Mean | 0.0 | 6.4 | 10.4 | 17.0 | 19.7 | 21.3 | 22.0 | 21.6 | 20.0 | 17.6 | 15.6 | 14.2 | 12.5 | 10.1 | 7.1 | 5.4 | 3.3 |
| SD | 0.0 | 4.3 | 5.4 | 8.0 | 8.5 | 8.3 | 6.8 | 7.0 | 6.0 | 5.3 | 4.6 | 4.1 | 3.4 | 2.9 | 2.1 | 1.8 | 1.0 |
| CV% | NA | 68.0 | 51.9 | 47.2 | 43.1 | 39.1 | 30.9 | 32.5 | 29.9 | 30.1 | 29.9 | 28.7 | 26.9 | 29.0 | 29.1 | 33.6 | 29.3 |
| Geo Mean | 1.0 | 4.4 | 8.5 | 15.2 | 17.9 | 19.7 | 20.9 | 20.4 | 19.0 | 16.7 | 14.9 | 13.5 | 12.1 | 9.6 | 6.8 | 5.1 | 3.2 |
| Geo CV% | 0.0 | 141.1 | 88.6 | 55.3 | 51.7 | 45.2 | 37.3 | 38.9 | 37.2 | 35.6 | 34.3 | 33.7 | 30.9 | 32.9 | 33.7 | 37.8 | 33.8 |

**2.4 Supplementary Table 7** Individual ratios of pharmacokinetic parameters of GP1707D07 in Period 2 compared to Period 1.

|  | C_max_(ng/mL) | | | AUC_0-t_(h*ng/mL) | | |
| --- | --- | --- | --- | --- | --- | --- |
| Subject ID | Period 1  GP681 alone | Period 2  GP681+itraconazole | Ratio  Period 2/Period 1 | Period 1  GP681 alone | Period 2  GP681+itraconazole | Ratio  Period 2/Period 1 |
| 301 | 16.0 | 20.4 | 1.3 | 759.4 | 1661.6 | 2.2 |
| 302 | 20.5 | 33.6 | 1.6 | 1247.3 | 2484.5 | 2.0 |
| 303 | 30.1 | 31.7 | 1.1 | 1364.5 | 2488.3 | 1.8 |
| 304 | 3.4 | 27.0 | 8.0 | 354.0 | 2757.8 | 7.8 |
| 305 | 11.2 | 14.5 | 1.3 | 739.8 | 1586.8 | 2.1 |
| 306 | 44.4 | 26.5 | 0.6 | 1770.2 | 2524.5 | 1.4 |
| 307 | 18.0 | 30.4 | 1.7 | 1069.8 | 2991.1 | 2.8 |
| 308 | 26.9 | 21.6 | 0.8 | 1199.3 | 2032.4 | 1.7 |
| 309 | 7.2 | 13.0 | 1.8 | 602.3 | 1353.9 | 2.2 |
| 310 | 15.5 | 20.9 | 1.3 | 1158.8 | 2127.2 | 1.8 |
| 311 | 12.0 | 9.9 | 0.8 | 728.2 | 1044.8 | 1.4 |
| 312 | 26.0 | 32.7 | 1.3 | 1488.0 | 2765.0 | 1.9 |
